# Blastomycosis Presenting as a Primary Tracheal Tumor: A Rare Presentation

**DOI:** 10.7759/cureus.31869

**Published:** 2022-11-24

**Authors:** Ahmad Rimawi, Karim Amireh, Traves Crabtree, Robert Robinson

**Affiliations:** 1 Internal Medicine, Southern Illinois University School of Medicine, Springfield, USA; 2 School of Medicine, American University of the Caribbean, Cupecoy, SXM; 3 Division of Cardiothoracic Surgery, Southern Illinois University School of Medicine, Springfield, USA

**Keywords:** tracheal tumor, fungal infections, bronchoscopy, hemoptysis, disseminated blastomycosis

## Abstract

Blastomycosis is a rare fungal infection that typically presents as a pulmonary infection. Systemic involvement of blastomycosis from the lungs commonly occurs in the skin and bones. Tracheal involvement is an unusual presentation of blastomycosis, which makes it a formidable diagnostic challenge. We herein report an unusual case of an 85-year-old man presenting with tracheal blastomycosis presenting as a primary tracheal tumor. We also highlight the challenges that were faced in the diagnosis of such an uncommon presentation. To the best of our knowledge, this is only the third occurrence of blastomycosis with tracheal involvement.

## Introduction

Blastomycosis is an uncommon fungal infection that most commonly occurs after inhalation of fungal spores from soil [1]. Blastomycosis is an infection known to be endemic to the Ohio and Mississippi river valleys but was reported in many other regions across the United States. It often presents as acute or chronic pneumonia and may cause minimal symptoms that resolve spontaneously [2]. Hematogenous spread, although uncommon, is a frequently reported complication of the disease. Extrapulmonary spread most commonly involves the skin, bones, and urinary system [3]. Tracheal involvement is an unusual presentation for blastomycosis that was only reported twice in the literature [4]. We report a case of tracheal blastomycosis presenting as a primary tracheal tumor.

## Case presentation

**Figure 1:**
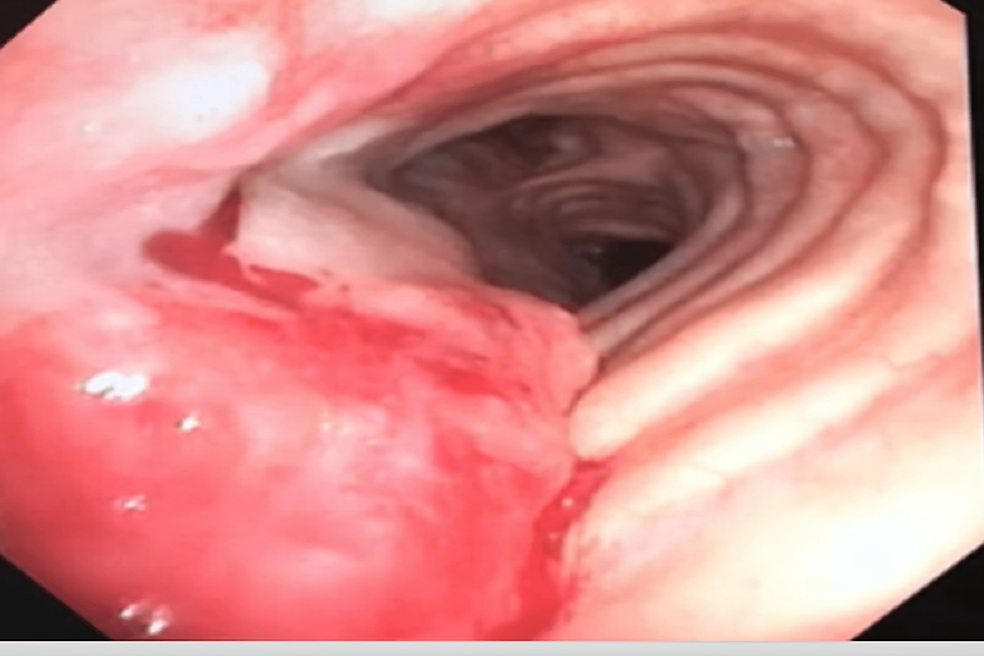
Bleeding tumor in the mid-tracheal region seen by flexible bronchoscopy.

The patient is an 85-year-old male with a past medical history of chronic obstructive pulmonary disease (COPD) requiring two liters of home oxygen and an extensive smoking history. He initially presented to the outpatient clinics after developing multiple episodes of epistaxis and coughing out blood for a two-week duration. Initial workup, including coagulation studies, complete blood count, basic metabolic profile, and chest x-ray, were all normal. He was referred to the otolaryngology clinic for further evaluation of epistaxis, and a nasal endoscopy was performed; inspection of the nasal cavity, sinuses, and larynx did not reveal any source of bleeding. After the preceding workup, the patient reported feeling blood coming out of his throat rather than the nasal cavity. He was then referred to the pulmonology clinic, and a decision was made to proceed with bronchoscopy to investigate a suspected pulmonary source of bleeding. A bronchoscopy was performed and revealed a 2 cm × 4 cm mid-tracheal bleeding tumor in the posterior tracheal wall that was friable and bleeding with contact. Given the friability of the lesion at that time, it was not biopsied due to a fear of excessive bleeding. A second bronchoscopy was performed that revealed the same 2 cm × 4 cm bleeding tumor in the mid-trachea (Figure 1), multiple laryngeal nodules and no lesions were visualized across the bronchial tree. Bronchoalveolar lavage was performed, and the return fluid was blood-tinged. The cytology of bronchoalveolar fluid showed no malignant cells and increased neutrophils with no organisms.

**Figure 2:**
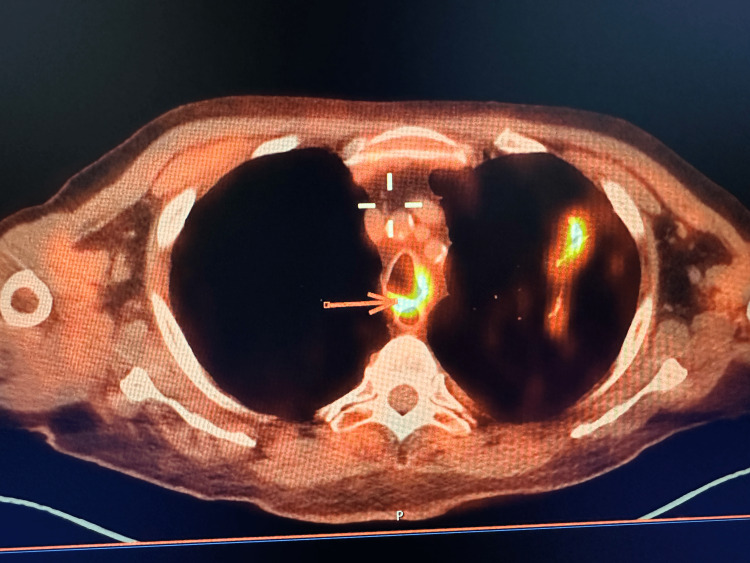
Positron emission tomography scan showing increased uptake in the mid-tracheal region.

**Figure 3:**
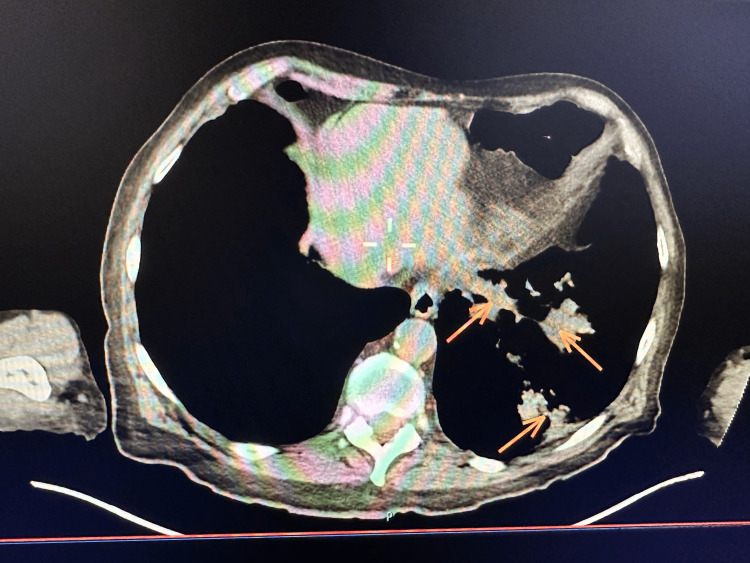
Computed tomography scan showing three pulmonary lesions in the left lower lobe of the lung.

**Figure 4:**
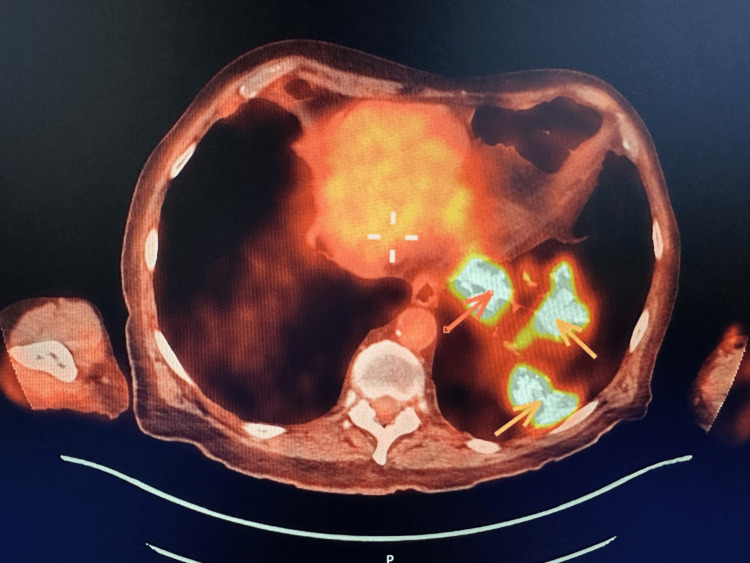
Positron emission tomography of the left lung showing three enhancing lesions.

A biopsy of the tracheal lesion was taken without significant bleeding and sent to the pathology lab. Nearly one week after the second bronchoscopy, the patient started to develop shortness of breath, cough, and fever that were gradually increasing in intensity. Given the patient’s extensive smoking history and the concerning appearance of his tracheal tumor, we had a very high suspicion of malignancy and decided to order a positron emission tomography/computed tomography (PET/CT) to investigate for possible metastasis. PET/CT showed multiple enhancing pulmonary lesions in the left lobe of the lung and an enhancing lesion in the mid-trachea (Figures 2, 3, 4).

Following the PET/CT scan, the biopsy results from the bronchoscopy came out and showed squamous epithelial inflammation and multiple fungi morphologically consistent with blastomycosis. Special stains, including Grocott’s methenamine silver stain, periodic acid-Schiff stain, and mucicarmine block C1 stain, were all used to confirm the presence of blastomycosis. A diagnosis of disseminated blastomycosis was given, and the patient was sent to the emergency department for hospital admission and further treatment. Upon admission, liposomal amphotericin B was started at a dose of 3 mg/kg every 24 hours for a total of two weeks, after which the patient stopped coughing out blood and his fever resolved. He was discharged from the hospital and followed up with the infectious disease outpatient clinic, where oral itraconazole was started at a dose of 200 mg twice daily for a total of 12 months for disseminated blastomycosis, which the patient is still completing. A follow-up CT scan of the chest was performed after two months of discharge and showed significant improvement (but not resolution) of the pulmonary lesions previously detected by PET/CT. The patient is still on antifungal treatment and is being monitored by the infectious disease outpatient clinic.

## Discussion

Blastomycosis is a rare infection that most commonly affects the lungs; direct inhalation of conidia from the soil is the primary route to developing an infection. In symptomatic cases, up to 40% of patients will develop extrapulmonary symptoms [1]. Although extrapulmonary involvement is common, tracheal involvement was rarely reported, with only two other cases in the literature [4]. In close proximity to the trachea, the larynx is the most commonly involved head and neck organ in blastomycosis [5]. Laryngeal involvement was studied across many regions in the United States and may reach up to 5% of all recorded cases [6]. While the trachea is in much closer proximity to the lungs (the primarily involved organ in blastomycosis), it had a much rarer involvement than the larynx. Extrapulmonary involvement in blastomycosis most commonly results from bloodstream dissemination. However, laryngeal involvement in blastomycosis was most presumed to result from direct inoculation of the laryngeal mucosa via inhalation [6]. We hypothesize that tracheal involvement follows the same route as laryngeal involvement, given the continuous tracts from the larynx to the trachea and the nearly identical mucosa.

In the previous two reports of tracheal blastomycosis, patients presented with symptoms of a pulmonary infection, including fever, sputum production, and cough [4,7]. Although our patient subsequently developed similar clinical features, his primary symptoms warranting investigation were hemoptysis and the presence of a solitary tracheal tumor. Blastomycosis was not on our differential diagnosis during the initial presentation, given its very low incidence and unusual tracheal involvement. Furthermore, the suspicion of malignancy in our patient was high given his old age, smoking history, and the absence of a common non-malignant differential. Establishing a biopsy-proven diagnosis of blastomycosis in our patient was of higher priority given the current guidelines of treatment. According to the American Society of Infectious Diseases, blastomycosis almost always requires treatment with antifungals, unlike histoplasmosis, which in many cases can resolve spontaneously [8].

## Conclusions

This case report presents a rare case of blastomycosis presenting as a primary tracheal tumor. To our knowledge, this is the third reported case of tracheal involvement in blastomycosis. Clinicians need to acknowledge that blastomycosis may have atypical presentations, especially when dissemination occurs. A very high suspicion is required to diagnose such cases, especially in highly endemic areas of the United States.
